# The Role of DNA Damage Response in Dysbiosis-Induced Colorectal Cancer

**DOI:** 10.3390/cells10081934

**Published:** 2021-07-29

**Authors:** Antonio Rivas-Domínguez, Nuria Pastor, Laura Martínez-López, Julia Colón-Pérez, Beatriz Bermúdez, Manuel Luis Orta

**Affiliations:** Department of Cell Biology, Faculty of Biology, University of Seville, Av. Reina Mercedes s/n, 41012 Seville, Spain; ardominguez@us.es (A.R.-D.); npastor@us.es (N.P.); lmlopez@us.es (L.M.-L.); colonperezjulia@gmail.com (J.C.-P.)

**Keywords:** DNA damage, microbiota, nutrition, ROS, bacterial toxins, inflammation

## Abstract

The high incidence of colorectal cancer (CRC) in developed countries indicates a predominant role of the environment as a causative factor. Natural gut microbiota provides multiple benefits to humans. Dysbiosis is characterized by an unbalanced microbiota and causes intestinal damage and inflammation. The latter is a common denominator in many cancers including CRC. Indeed, in an inflammation scenario, cellular growth is promoted and immune cells release Reactive Oxygen Species (ROS) and Reactive Nitrogen Species (RNS), which cause DNA damage. Apart from that, many metabolites from the diet are converted into DNA damaging agents by microbiota and some bacteria deliver DNA damaging toxins in dysbiosis conditions as well. The interactions between diet, microbiota, inflammation, and CRC are not the result of a straightforward relationship, but rather a network of multifactorial interactions that deserve deep consideration, as their consequences are not yet fully elucidated. In this paper, we will review the influence of dysbiosis in the induction of DNA damage and CRC.

## 1. Human Microbiota

Microbiota is defined as the group of microorganisms that naturally inhabit the body of pluricellular organisms. This term includes a highly variable and complex community of fungi, viruses and bacteria that occupies specific niches in healthy organisms [1,2]. In the human body, the number of microorganisms is approximately the same as that of human cells, which is indicative of their significance in human biology [3,4].

Microbiota plays a transcendental role in physiological functions. This community fulfills metabolic, neuronal and immune requirements including the establishment of a protective barrier. Nevertheless, in dysbiosis conditions, it is not that the number of microorganisms may decrease but that the diversity of colonizers changes, with a major impact on homeostasis. This situation can contribute to the development of autoimmune, or inflammatory diseases and cancer [4,5,6].

The term cancer includes a group of diseases characterized by uncontrolled cell proliferation. Its development depends not only on genetic predisposition but also on environmental factors. In this particular point, dysbiosis might play important roles in carcinogenesis and influence their therapy [7,8,9].

Recently, several studies have reported that a direct secretion of enzymes or molecules from microbiota can influence the activation of NFkB (nuclear factor kappa-light-chain-enhancer of activated B cells), apoptosis pathways or cytoskeleton reorganization. Moreover, chemically modified nutrients from diet act as microbiota modulators boosting ROS/RNS production, toxins and the onset of CRC process [10,11,12].

Here, we will review the incidence of gut bacterial dysbiosis in CRC development as well as the mechanisms involved, with special emphasis on those that cause DNA damage.

## 2. DNA Damage and Cancer, Old Friends

A well-known feature of cancer cells is genomic instability. Indeed, DNA damage is responsible for point mutations or chromosome rearrangements frequently found in transformed cells. Chronic inflammation conditions, as those involved in dysbiosis, may promote environmental conditions that favor cancer development through induction of DNA damage [13,14,15]. 

DNA can be damaged by endogenous or exogenous sources. Endogenous sources include ROS/RNS, toxic products from cellular metabolism or disturbances in DNA replication, i.e., DNA replication–transcription conflicts. On the other hand, ionizing radiation, UV light and many toxic chemicals used in therapy are exogenous threats to DNA integrity.

DNA Single-Strand Breaks (SSBs) or base damage can be easily found in cells spontaneously as a consequence of the action of ROS and RNS. In this sense, a Base Excision Repair mechanism (BER) can restore the original DNA sequence [13,16]. In the first step of this process, damaged bases are recognized and excised by DNA glycosylases. Monofunctional DNA glycosylases such as Uracil DNA Glycosylase (UNG) create only an abasic site. However, bifunctional glycosylases, such as OGG1, also create a nick on the 3′ side of the abasic site [16]. The resulting apurinic/apyrimidinic (AP) site or the nicked DNA are the targets for AP endonuclease (AP-1), which breaks the phosphodiester bond to create an SSB [16]. Normally Pol β refills the gaps and nicks are resealed by DNA ligase 1 or ligase 3 [16].

The relationship between BER and Poly (ADP-ribose) polymerase-1 (PARP-1) has been largely discussed. PARP-1 is reported to be a sensor of SSBs [13,16,17] that arise either directly or as intermediates of BER [13,16,17]. Indeed SSBs are protected from degradation by PARP-1 which additionally potentiates the recruitment of repair factors [16]. However, the involvement of PARP-1 as a member of BER has resulted in controversy over the years.

The Mismatch Repair (MMR) pathway detects and removes DNA base-pair mismatches and inappropriate nucleotide insertions/deletions (INDELs) that arise during DNA replication. There are two important protein complexes involved in MMR, namely MutS and MutL. MutS has two isoforms; the first (MutSα) is constituted by MSH2 and MSH6, and the second one (MutSβ) by MSH2 and MSH3. MutL presents three isoforms namely MutLα (MLH1/PMS2), MutLβ (MLH1/MLH2) and MutLγ (MLH1/MLH3). It was shown that mutations in one-off MSH2 or MLH1 can affect the entire system [18,19,20]. Mechanistically, the mismatch is recognized by MutS, then the endonuclease MutL and the exonuclease EXO1 are recruited. Once resection in the appropriated DNA strand is finished, polymerase δ and DNA ligase I repair the excised region [21,22]. 

Microsatellite regions are short sequences of 1 to 6 base pairs, repeated in tandem and present all through the genome. Due to their nature, they are especially prone to induce replication errors, which are normally repaired by MMR. In this sense, any inactivating mutation in the MMR genes mentioned above results in a hyper-mutant phenotype known as microsatellite instability (MSI), due to a defective MMR system (dMMR) [20,21,23].

Nucleotide Excision Repair (NER) repairs bulky- or helix distorting-DNA lesions. Depending on how these injuries are detected, NER is classified into Global- (G-NER) or Transcription-Coupled NER (TC-NER). While G-NER is able to recognize lesions all through the genome, TC-NER is initiated by the blocking of RNA polymerases by DNA damage. The subsequent steps are identical in both branches: DNA is then opened, a single-strand DNA (ssDNA) region of approximately 24–30 base pairs is generated, subsequently refilled by replication polymerases and ligated by ligase I [24].

The DNA Damage Response (DDR) coordinates the signaling and repair of Double-Strand Breaks (DSBs) and long stretches of ssDNA with the cell cycle checkpoints [25]. This is carried out by three phosphoinositide 3-kinase (PI3K)-related serine-threonine kinases, namely DNA-dependent protein kinase (DNA-PK), ataxia telangiectasia-mutated kinase (ATM) and ataxia telangiectasia and Rad3-related protein (ATR) [25,26]. 

ssDNA stretches accumulate when cells suffer replication stress, as intermediates of the NER pathway and after the resection of DSBs. They are detected by ATR, which has a predominant role in phosphorylating and activating CHK1. The resulting ATR-CHK1 complex mediates various cell responses that include S and G2/M checkpoints that facilitate DNA repair [27]. Additionally, ATR promotes Homologous Recombination (HR), regulates proper replication initiation and faithful chromosomal segregation [27,28].

The most difficult DNA lesion to repair is a DSB. One single unrepaired DSB can induce cell death when essential gene is affected [13]. The MRE11-RAD50-NBS1 (MRN) complex recognizes the DSB attracting ATM. ATM phosphorylates several proteins that will mediate cell cycle arrest and DNA repair [25]. In this sense, DNA-PK and H2AX histone are phosphorylated and hence activated by ATM [29]. Phosphorylated H2AX (γ-H2AX) will recruit more ATM molecules together with DNA repair factors [25].

DSBs are repaired by two major pathways namely HR and Non-Homologous End Joining (NHEJ) [25,30,31]. While the former needs the presence of a homologous sequence (usually a sister chromatid) to faithfully repair a DSB, the latter reseals directly the two broken edges, which sometimes may put in place new mutations [25,30,31]. As a consequence, NHEJ is active during all the cell cycles but HR operates mainly during S and G2 phases.

One-ended DSBs are formed by either collision between replication forks and SSBs or after the processing of stalled forks, and are preferentially repaired by HR. However two-ended DSBs can be repaired by both systems [30].

**Figure 1:**
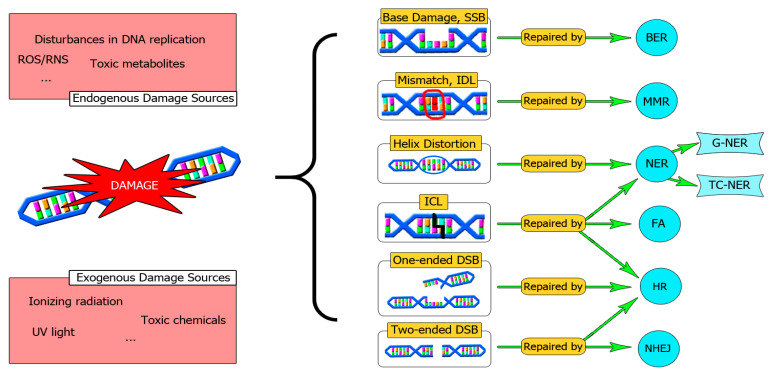
An overview of the main DNA repair systems. DNA can be damaged by exogenous or endogenous agents. A DNA repair defect can be a source of mutations that drive malignancy progression. Sometimes there is a cooperation between different repair pathways to solve complex DNA lesions, such as ICLs. In this sense, a fine-tune cooperation between HR and NER orchestrated by FA is required. SSBs: Single Strand Breaks, BER: Base Excision Repair, IDL: Insertion-Deletion Loops, MMR: Mismatch Repair System, NER: Nucleotide Excision Repair, TC-NER: Transcription coupled-NER, G-NER: Global-NER, DSBs: Double-Strand Breaks, NHEJ: Non-Homologous End Joining, HR: Homologous Recombination, ICL: Interstrand Cross Links, FA: Fanconi Anemia Pathway.

A summary of the main DNA repair pathways is depicted in Figure 1.

## 3. Colorectal Cancer: The Origin

Although CRC incidence ranks in the third position, and second in terms of mortality worldwide, only a small percentage is attributable to hereditary factors, most of them appear in transitioned countries (4-fold higher) and it is due to an inadequate lifestyle [32].

### 3.1. The Poisoned Inheritance

Between 2–5% of CRC cases have a hereditary component. The Familiar Adenomatous Polyposis (FAP) is present in approximately 1% of CRC cases and is caused by inherited *APC* mutations (an early event that facilitates the adenoma–carcinoma transition via WNT activation) [33,34]. Other examples of CRC-associated inherited mutations are *MUTYH*-Associated Polyposis [35], Peutz-Jeghers Syndrome [36] or Serrated Polyposis Syndrome [36]. Of note, hereditary non-polyposis colorectal cancer (HNPCC), also known as Lynch Syndrome (LS), is characterized by mutations that inactivate the MMR pathway and represent 3–4% of all CRC cases [33,34].

Nevertheless, MMR pathway defects are abundant also in sporadic CRC. Indeed, *MLH1* appears inactivated by bi-allelic promoter methylation in 13–16% of sporadic cancers driving to microsatellite instability (MSI). A defect in MMR has been associated with mutations in key cellular signaling genes, such as *BRAF*, that has been linked to the onset of CRC [20,21,23,37]. Furthermore, a defective MMR was associated with the generation of neoantigens that promote cell survival against the immune system [20].

In other cases, cancer cells are microsatellite-stable but chromosomally unstable (CIN) showing mutations in *APC*, *TP53*, *KRAS*, *SMAD4*, and *PIK3CA* (around 84% of sporadic CRC) [20,33,38,39]. 

### 3.2. Environment Is the Key

A large number of studies confirm the evidence that environmental factors rather than inherited genetic dysfunctions operate in the development of most CRC cases [40]. 

A key event in the development of CRC is the tumourigenic atmosphere caused by the loss of the epithelium barrier. The breakage of the epithelial layer favors the contact of bacterial epitopes with immune cells in lamina propria, triggering an exacerbated immune response that perturbs colon homeostasis [41]. As a result, this proinflammatory microenvironment promotes the detachment and mobilization of epithelial cells favoring a dysplasia state [42].

In this aberrant scenario, leukocytes deliver proinflammatory cytokines such as IL-1β, IL-6 and TNF-α. IL-6 mediates the release of molecules that promote proliferation, angiogenesis and cell survival [43,44,45]. IL-1 activates the RAS/MAPK pathway, drives NFkB downstream genes activation, favors autophagy suppression, tumour cell migration-invasion and aggressiveness [46,47,48,49]. Furthermore, IL-1 was associated with a decrease in epithelial E-cadherin, which increases the permeability of the epithelial barrier and favors tumour invasion [50,51,52]. 

TNF-α is a key regulator of ROS and RNS signaling. ROS link to several cellular processes as part of signaling pathways. NFκB regulates genes that modulate the amount of ROS and, as a feedback loop, ROS might have a stimulatory or inhibitory role in NFκB signaling [53,54]. In a proinflammatory process, ROS levels might degenerate into a toxic effect in neighboring cells, exerting a harmful effect on lipids, proteins, and especially nucleic acids. Long-lasting elevated concentrations of ROS can promote cellular transformation by inducing DNA damage, cell growth, angiogenesis and metastasis [55,56]. 

High levels of ROS lead to genetic instability and SSBs because of oxidation of pyrimidines and purines and induction of alkali-labile sites [57]. The nucleotide with the highest oxidation potential is guanine, giving rise to 8-oxo-7,8-dihydro-2′-deoxyguanosine (8-oxoG). Incorporated 8-oxoG can be repaired either by BER or MMR, but if it is left unrepaired C:G > A:T transversion mutations can appear after replication [58]. BER glycosylases involved in the repair of incorporated 8-oxoG are OGG1 (in 8-oxoG:C base pairs) and MUTYH (in 8-oxoG:A pairs) [16,58]. An excessive number of SSBs, intermediates of BER and MMR, will induce replication- or transcription-coupled DSBs that will finally end in chromosome aberrations and cell transformation [58].

Effector immune cells in organs with chronic inflammation release abnormal levels of nitric oxide (NO), which may alter the physiological function and normal metabolic state, leading to nitrosation of amines [59]. NO is synthesized by several isoforms, being inducible NOS (iNOS), which exhibit greater NO output in immune responses [60]. iNOS uses NADPH, L-arginine (L-Arg) and oxygen to generate NO and L-citrulline (L-Cit). When oxygen reacts to the NO surplus produced during the inflammatory process, generates nitrogen oxides byproducts such as peroxynitrite, N_2_O_3_ and N_2_O_4_, which are powerful nitrosating agents [61].

Indeed, RNS converts secondary amines into activated N-nitrosamine intermediates, which are mutagenic and interact with DNA repair enzymes and transcription factors such as NFkB (see below) [62,63]. Despite the fact that most endogenous nitrosamines are produced by the stomach, different studies have shown nitrosamine formation due to amine production by intestinal bacteria, leading to an increased risk of CRC development [64,65,66].

Diet-related chronic intestinal inflammation is a proven risk factor for CRC due to colonization by pathogenic microorganisms, which serve to provide pro-tumour immunity, exposure of intestinal cells to their mutagenic metabolites and hence potentiate inflammation. These microorganisms include Colibactin-producing *Escherichia coli*, enterotoxigenic *Bacteroides fragilis* and *Helicobacter hepaticus* among others [67].

It was recently reported that tissue damage or, chronic inflammation induced by such bacteria species, induce oxidative stress and hence 8-oxoG and DSBs [58]. Furthermore, antioxidants such as vitamin C are able to reduce DNA damage and tumourigenesis in this context [58]. In a mouse model for LS, 8-oxoG and DSB resulted higher in MMR defective animals, but antioxidants failed to prevent tumourigenesis [58].

In the next points, we will review more environmental factors involved in CRC development under a DNA damage perspective, such as microbiota-modified metabolites from diet or bacterial toxins.

## 4. DNA Damage Induced by Metabolites from Diet

Nutritional habits seem to modulate microbiota diversity and hence influence colorectal tumourigenesis [68,69,70]. Healthy diets such as vegan, Mediterranean, or Japanese diets have demonstrated protection against the development of several cancers, besides a reduction in fatality associated with them [71]. It is well accepted that high fiber food protects against CRC, on the other hand, high amounts of red meat, saturated fat and processed food favor its development [40,72,73,74]. Some bacterial strains present in healthy microbiota are *Bacteroidetes* (Gram-negative) and *Firmicutes* (Gram-positive) with lower amounts of *Actinobacteria* and *Verrumicrobia* [75]. In contrast, certain strains of *Bacteroides fragilis* and *Escherichia coli* (carrying PKs islands), *Streptococcus gallolyticus*, *Enterococcus faecalis* and *Fusobacterium nucleatum* are abundant in CRC patients [49,67,76].

**Figure 2:**
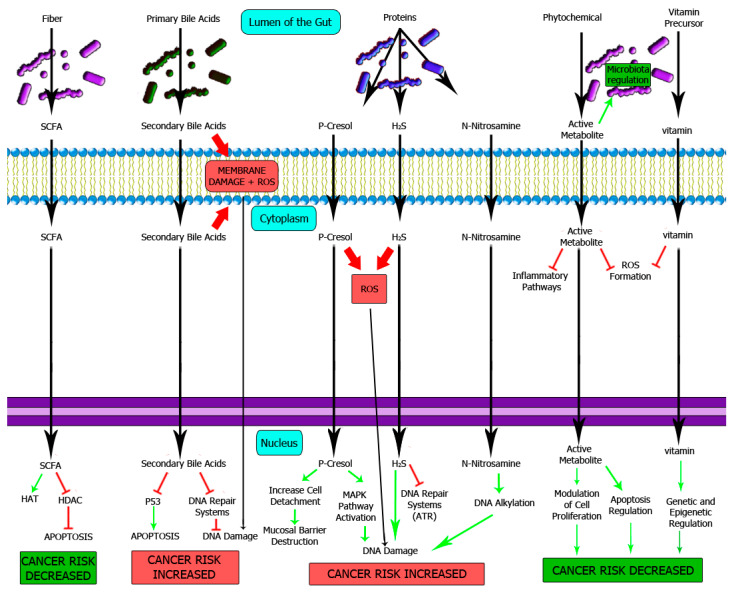
Influence of microbiota-processed nutrients on colon epithelium homeostasis. The importance of normal microbiota has been extensively studied, with a major impact on the protection against CRC. For instance, short-chain fatty acids are tumour suppressor metabolites produced by healthy typical microbiota that protects normal epithelium against neoplasia through an epigenetic mechanism. On the other hand, an excess of oncometabolites such as p-cresol, secondary bile acids or N-nitrosamines will favor mutagenesis and cancer development. SCFA: Short-chain fatty acids, HAT: Histone acetyltransferase, HDAC: Histone deacetylase.

Most nutrients are absorbed in the small intestine; nevertheless, complex carbohydrates, protein-derived compounds and bile acids might reach the large intestine [40]. Once there, microbiota metabolizes those compounds giving rise to “oncometabolites” and “tumour suppressor metabolites” with immune, epigenetic and genotoxic consequences [72]. The proposed mechanisms for these compounds are summarized in Figure 2.

### 4.1. Metabolites from Carbohydrates 

Butyrate, propionate and acetate are short-chain fatty acids (SCFA) that result from dietary fiber fermentation by microbiota [77]. These compounds may suppress tumour development through several mechanisms [40]. The main SCFA producers are *Bacteroidetes, Clostridium, Eubacteria and Roseburia* [40,74,78], which were found to be diminished in CRC patients [78].

The importance of butyrate in the intestinal epithelium is well documented [40,77]. Butyrate is the main energy source for enterocytes, increases mucin production and induces the protein expression of claudins and occludines, which participate in the epithelial barrier [77]. Epithelial impermeability is important to avoid the translocation of bacteria or their metabolites to lamina propria, preventing inflammatory episodes. 

In a healthy colon, butyrate induces cell proliferation due to its role as an energy supplier but also decreases cancer risk through epigenetic regulation [40]. Here, butyrate is metabolized into acetyl-CoA, which stimulates histone acetyltransferase (HAT) activity [79]. 

On the other hand, CRC is characterized by a high protein expression of HDAC (Histone deacetylase) [40,80], which drives cells to profound epigenetic changes [80]. Due to the Warburg effect, transformed cells prefer glucose to butyrate as energy source. Subsequently, there is an accumulation of butyrate in neoplastic epithelium, which triggers apoptosis via HDAC inhibition [40,73,79].

### 4.2. Metabolites from Fat and Bile Acids

Bile acids are secreted into the small intestine from the gallbladder where they emulsify dietary fats [81]. Primary bile acids such as cholic and chenodeoxycholic, are synthesized by the liver from cholesterol and conjugated with glycine or taurine which increases its emulsifying properties [81].

Most of the bile acids are recycled into the enterohepatic circulation. Nevertheless, a residual fraction may remain in the large intestine where it is converted by *Bacteroides* and *Bilophilia* into secondary bile acids such as deoxycholic and lithocholic [82].

Deoxycholic and lithocholic cause ROS and membrane damage in enterocytes. Subsequently, arachidonic acid is released and converted by Lipoxygenase (LOX) and Cycloxygenase 2 (COX-2) enzymes into inflammatory prostaglandines and ROS overproduction [9].

Furthermore, secondary bile acids have an inhibitory effect on DNA repair systems, leading to an increment of mutated cells with a marked genetic instability, characterized by the induction of SSB, DSB and aneuploidy [82,83]. DNA repair systems downregulated by secondary bile acids are HR, NER, NHEJ and MMR. Furthermore, levels of ATM are reduced as well as OGG1 and MUTYH glycosylases [82,83]. 

Additionally, it was reported that deoxycholic acid induces proteasomal degradation of p53 [84] and activates survival and proliferative pathways such as Wnt/β-catenin [83], PKC [85] and NFkB [85], which egress apoptosis-resistant clones [82,83].

### 4.3. Metabolites from Proteins 

Protein degradation by colon microbiota has been extensively studied [40,81,86]. Colon bacteria break down undigested peptides, digestive enzymes, mucin and cell debris from the small intestine [40,81,86]. As a result, fatty acids, short peptides and amino acids are generated. However, many toxic compounds are also released such as amines, nitrates, nitrites, N-nitrosamines, hydrogen sulfide (H_2_S), p-cresol and ammonia [40]. Protein fermentation by microbiota is higher at the distal part of the large intestine, where these metabolites are found at larger concentrations [86,87].

#### 4.3.1. p-Cresol

When aromatic amino acids, such as tyrosine, tryptophan and phenylalanine ferment, a wide variety of phenolic and indolic compounds are generated. For instance, tyrosine fermentation generates p-cresol reaching up to 0.5 mM concentration in human feces [87,88]. Most of the p-cresol is absorbed by enterocytes and later on, metabolized by the liver and excreted in urine [86,87].

Andriamihaja and colleagues studied the deleterious effects of millimolar concentrations of p-cresol on the human adenocarcinoma cell line HT-29 Glc ^−/+^ [87]. They found that p-cresol at 0.8 mM diminished cell proliferation due to an increase in cell detachment and S-phase delay [87]. Potentially, this detachment could implicate the disruption of the epithelial barrier in vivo and causes colon inflammation. P-cresol concentrations above 1.6 mM were genotoxic as measured by the γ-H2AX foci assay. Additionally, p-cresol had a negative effect on the mitochondrial respiratory chain with subsequent anion superoxide (ROS) production. According to the authors, the observed toxicity is independent of DNA damage induction [87].

Recently, a report strengthens the hypothesis that colon microbiota-derived p-cresol behaves as a genotoxic agent at physiological concentrations [75]. Bacterial cultures from human fecal inoculums were grown with a high tyrosine supplement and supernatants were used to treat HT29 and Caco-2 cells for 24 h. According to the data, p-cresol could serve as a great predictor of genotoxicity in enterocytes. Cell cycle was arrested in S phase at 0.5 mM, however, this effect was not evident at higher concentrations [75]. Authors explain that low doses of p-cresol induce tumour growth [89], though; this effect is masked at higher concentrations by DNA damage-induced G0/G1 and G2/M checkpoints [75].

#### 4.3.2. H_2_S

Another important residual metabolite from sulfate-reducing bacteria is H_2_S [40,81,90]. Classically this molecule was considered as a toxic molecule, although in recent decades, it is believed that H_2_S behaves as a signaling molecule in physiological processes such as cell proliferation, apoptosis, inflammation, hypoxia, neuromodulation and cardioprotection [91]. In mammals, the production of H_2_S results from the enzymatic action of cystathionine beta-synthase (CBS), cystathionine gamma-lyase (CSE) and 3-mercaptopyruvate sulfurtransferase (3-MST) [91].

Regarding to CRC, H_2_S has been described to have pro- and anticancer effects. Endogenously produced H_2_S or low H_2_S treatments can maintain or promote cancer growth while high exposures exhibit anticancer effects [92]. Endogenously CBS-produced H_2_S can promote angiogenesis and maintain cellular bioenergy in colon cancer cells [92]. Furthermore, at 24 h of exogenous exposure to 50–200 µM of NaHS (a donor of H_2_S), can accelerate cell cycle progression by decreasing the levels of p21 and increasing the levels of S phase cells [92,93]. However, relatively high concentrations of exogenous H_2_S can have a growth-suppressive effect. For instance, 1 mM of NaHS for 12–24 h upregulates the protein expression of p21 in human colon cancer cells [92,94].

Another main issue is autophagy. Autophagy is a catabolic process that involves the lysosomal digestion and subsequent recycling of internal components [95]. Concerning cancer progression, autophagy has a dual role. Initially, in pre-malignant lesions, autophagy prevents cancer development but if cancer is well-established, autophagy facilitates tumour survival and growth [95]. Recently, it was shown that endogenous levels or exogenous treatments with H_2_S in several hepatocarcinoma cell lines could present opposite effects on autophagy [96].

In reference to DNA damage induction, H_2_S was reported to be genotoxic for enterocytes and has been associated with ulcerative colitis and CRC [40,81,90,97,98]. H_2_S, at doses found in human colon (250 µM), induced DNA damage to CHO cells [97]. Similar data was found when the HT29-Cl.16E colon cell line was assayed with higher H_2_S concentrations [97]. In this case a modified alkaline comet assay, in which DNA repair was inhibited by hydroxyurea and a chain terminator (Ara-C) was used [99]. Taken together, a DNA repair defect and the presence of H_2_S arguably predispose enterocytes to genomic instability in a cancer progression context [97]. 

Later on, it was reported that H_2_S induced-DNA damage was based on a free radicals production mechanism [98]. The exposure to H_2_S rapidly increased the NADPH/NADP ratio through inhibition of mitochondrial respiratory chain in the non-transformed rat intestinal cell line IEC-18 [100,101]. The electron transport chain defect observed could be responsible for the generation of genotoxic free radicals.

Additionally, it was found that H_2_S induced DNA damage in a colon non-transformed human cell line (FHs 74 Int) at doses that can be found in large intestine [90]. Doses lower than 500 µM were genotoxic and induced changes in gene expression patterns without showing cytotoxic effects. Indeed, pro-inflammatory *COX-2* expression was approximately 8-fold upregulated after 30 min exposure [90]. The expression of several genes related to the DNA damage response was also altered. For instance, *GTF2H1*, belonging to multimeric transcription factor II H (TFIIH), which is involved in NER, and *XRCC6*, linked to NHEJ were upregulated within the first 30 min. However, *RAD51* (HR) and *MLH1* were downregulated after 4 h exposure [90].

A high protein expression of COX-2 was linked to transformed epithelial cells and activated macrophages in CRC [102,103]. Activation of the NFkB pathway and the subsequent synthesis of proinflammatory cytokines has also been reported in monocytes exposed to H_2_S [104]. 

In 2019, Chen and coworkers showed how H_2_S regulates ATR levels and its phosphorylation [28]. The presented data show how ATR orchestrates the DDR induced by H_2_S. Indeed, cells carrying *ATR* mutations showed DNA damage after low H_2_S exposure, and were hypersensitive to higher concentrations [28]. However, a complex regulatory mechanism between ATR and H_2_S was postulated [28]. First, ATR inversely regulates enzymes involved in H_2_S synthesis and hence H_2_S concentration. Second, high H_2_S concentrations suppress ATR phosphorylation at serine 435 (ATR-pS435) while low levels increase it [28]. Of interest, PKA-mediated ATR phosphorylation at serine 435 is required to promote NER and reduces mutagenesis via ATR-XPA complex formation [105].

#### 4.3.3. N-Nitrosamines

N-nitrosamines are organic molecules derived from protein fermentation. These compounds result from the combination of amines and nitrates. N-nitrosomorpholine, N-nitrosodimethylamine and N-nitrosopyrrolidine are the most relevant compounds in this group [40].

N-nitrosamines require metabolic activation by cytochrome P450 to wield their carcinogenic effect [106,107]. Then, nitrosamines are α- and β-hydroxylated giving rise to end products that can finally alkylate nucleophilic sites of DNA. Consequently, mutagenic alkali-labile adducts are generated, leading to abasic site formation and DNA strand breaks that can be detected by alkaline comet assay [106,107,108]. 

Moreover, it was previously reported that N-nitrosamines induce free radicals and therefore oxidized bases [106,107,108,109,110]. In this context, it was demonstrated in vitro that neutrophil activation may generate carcinogenic nitrosamines [65].

#### 4.3.4. Ammonia

To our knowledge, there are no reports that deepen in the analysis of DNA damage in enterocytes exposed to high ammonia concentrations. Ammonia caused p53 activation, p21 upregulation, mitochondrial dysfunction, ROS generation, DNA damage and cellular senescence in astrocytes, neurons and hepatic endothelial cells from hepatic encephalopathy patients [111,112]. In epithelial cells from mammary bovine glands, high ammonia concentrations gave rise to the same effects described above [113]. 

High ammonia concentrations reduce the absorptive capacity and survival rate of the enterocytes. This scenario promotes mucosal turnover, inflammation and fragility of the epithelial intestinal barrier [86].

### 4.4. Phytochemicals and Vitamins

Phytochemicals are micronutrients synthesized by plants and abundant in fruit, vegetables, legumes, tea or wine, highly beneficial to human health [114]. Due to their complexity, 95% of phytochemicals are absorbed and transformed into more active secondary metabolites by colon microbiota [115]. For instance, soy isoflavones such as daidzein or genistein can be differentially metabolized by microbiota giving alternative secondary metabolites [116,117].

Flavonoids are the largest group of phytochemicals. This group includes isoflavones, anthocyanins and catechins between others. 

The anticancer properties of isoflavones and their derivatives have been extensively studied. They are anti-inflammatory and antioxidant molecules that interfere in several cell signaling pathways such as NFkB, AKT or MAPK/ERK, inhibiting cancer growth [117,118]. 

Anthocyanins are flavonoids with anti-inflammatory, anti-oxidative and anti-cancer properties [119,120,121]. They modulate bacteria involved in CRC development, by inhibiting the propagation of *Helicobacter pylori* or promoting the growth of *Bifidobacterium* spp. and *Lactobacillus-Enterococus* spp. [122]. Additionally, these compounds are able to modulate the oxidative stress by blocking the phosphorylation of NFkB, which is one of the main causes of DNA damage, and downregulating *TNFα*, *COX2* and *iNOS* mRNA expression [123]. 

Flavonoids commonly named catechins are antioxidants and anti-inflammatory molecules. The underlying mechanisms comprise the inhibition of ROS, hypoxia and NFkB signaling cascades. In addition, catechins modulate COX2, block of the epidermal growth factor receptor (EGFR) and insulin-like growth factor receptor-1 (IGFR-1) signaling pathways [124]. 

Green tea catechins modify gut microbiota composition and protect against CRC. An elevated number of bacterial SCFA-producing strains, reduced *Fusobacterium* spp. and increased FIR/BAC (*Firmicutes* to *Bacteroidetes* ratio) ratio were reported [125]. Nevertheless, if catechins concentration is high enough, they behave as pro-oxidant elements generating ROS, DNA damage as well as MMPs production. Furthermore, inhibition of Topoisomerases I and II, which induce DNA damage, have been reported [126,127]. Indeed, catechins, in a dose-dependent manner, increase the yield of endoreduplicated cells, a topoisomerase II dysfunction marker [128]. 

Vitamins are essential organic elements for proper homeostasis. It is widely known that colon microbiota plays an important role in vitamin acquisition. Some bacteria strains can synthesize vitamins of K and B groups establishing another vitamin absorption source. Dysbiosis changes microbiota diversity and hence vitamin acquisition by colon may result altered [81].

Low levels of folate (vitamin B9) were associated with different types of cancer (colon, lungs, breast, brain, etc.) in adults, as well as cognitive deficiencies in babies. The underlying mechanism involved is linked to DNA synthesis, repair, and methylation. S-adenosyl methionine (SAM) donates methyl groups to DNA methyltransferases (DNMTs) and complete the DNA methylation process. When folate levels are low, SAM concentration is reduced, leading to DNA hypomethylation that links to proto-oncogenes mRNA expression [129]. 

Moreover, low folate conditions alter the purine-pyrimidine balance, giving rise to collapsed replication forks and hence one-ended DSBs [130]. Besides, vitamin B9 deficiency inhibits the methylation of dUMP to dTMP, which causes massive uracil incorporation into DNA. BER could result overwhelmed and then high amounts of SSBs and chromosomal breaks are generated [131]. 

Niacin or vitamin B3 is the precursor of nicotinamide adenine dinucleotide (NAD) and nicotinamide adenine dinucleotide phosphate (NADP). These coenzymes are cofactors in almost all metabolic processes, regulating PARP and sirtuins, among others, which are relevant for genetic and epigenetic regulation [132]. 

A deficiency in niacin unbalances the NAD^+^/NADH ratio disrupting a large number of processes including DNA repair. Genetic instability and increased risk of cancer development are frequently associated with low levels of niacin, as PARP requires the presence of NAD^+^ to efficiently repair DNA damage [132].

## 5. Microbiota Genotoxins

**Figure 3:**
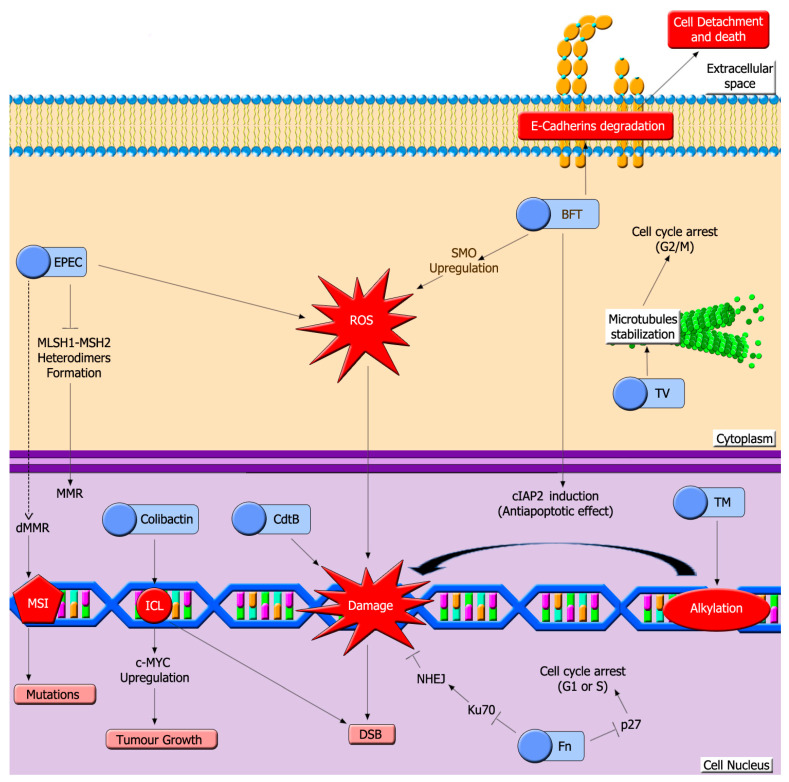
Bacterial toxins induce a high variety of DNA lesions in colon epithelium. A hallmark of colon dysbiosis is the growth of pathogenic bacteria that release toxins that induce DNA damage. Toxins depicted here can damage host DNA through different mechanisms, such as induction of interstrand crosslinks (ICL), generation of ROS, DNA alkylation or inhibiting MMR. EPEC: Enteropathogenic E. coli toxin, CdtB: Cytolethal distending toxin, BFT: Bacteroides fragilis toxin, Fn: Fusobacterium nucleatum toxins, TV: tilivalline, TM: tilimycin, MMR: mismatch repair system, dMMR: defective mismatch repair, MSI: microsatellite instability, DSB: double strand break.

Dysbiosis conditions and perturbed microbiota may include pathogenic strains that synthesize DNA damaging toxins (Figure 3) [133]. 

### 5.1. Colibactin

Colibactin is a genotoxic compound produced by some *E. coli* strains that can induce DSB, chromosomal aberrations and G2/M cell cycle arrest [134].

Three non-ribosomal peptide megasynthases, three polyketide megasynthases, two hybrid megasynthases and some accessory proteins are responsible for Colibactin synthesis as a propeptide [134]. To become active, the propeptide is processed by an inner-membrane-bound peptidase called Colibactin peptidase (ClbP) that cleaves acyl asparagine residues located in the N-terminus [135].

Active Colibactin can form interstrand cross-links (ICL) with DNA. These structures block replication forks and are processed into one-ended DSB via Fanconi Anemia Repair Pathway (FA) and finally repaired by HR (Figure 1). Indeed, Bossuet-Greif and coworkers found that γ-H2AX foci (a DSB marker) colocalized with FANCD2 (a FA marker) foci after Colibactin exposure [136]. In agreement with that, Colibactin induced an ATR-mediated replication stress response [136]. 

NHEJ deficient cells resulted hypersensitive to Colibactin, so apparently, two-ended DSB might be induced [137]. Herzon et al. deciphered more about the nature of Colibactin-induced DNA damage. They concluded that Colibactin induces N3-Adenine alkylations that are depurinated by BER into AP sites, promoting a SSB in each DNA strand and finally DSBs are formed [15,138,139]. 

This genotoxin promotes colon tumour growth by inducing a senescent cell phenotype that secretes growth factors. The mechanism is based on an up-regulation of c-MYC protein levels after DNA-damage induction. c-MYC increases microRNA-20a-5p expression that blocks *SENP1* mRNA translation [140]. This situation triggers an accumulation of sumoylated-P53. Therefore, the transcriptional activating function and DNA binding capacity of P53 will be abrogated [141]. Most likely sumoylated P53 enhances a senescent cellular state [142].

### 5.2. Toxins Generated by EPEC

Enteropathogenic *E. coli* (EPEC) can settle in the host’s gut epithelium through close interaction with intimin adhesion protein and disrupts MMR [143,144].

EPEC effector proteins may cause increased host mutations by depleting the MLH1 and MSH2 protein pool while their transcription is enhanced [145]. The underlying mechanism is mediated by ROS production and could disrupt MLH1 and MSH2 heterodimers formation. This mechanism is not enough to inhibit MMR completely, but Map and EspF proteins can totally block MSH2 [145,146]. MMR dysfunction increases spontaneous mutations that can affect tumour suppressor genes. This could explain the axis between chronic EPEC infections and CRC [144,146].

### 5.3. Cytolethal Distending Toxins (Cdt)

Cdt are a family of cytotoxins produced by different bacterial strains including *Helicobacter hepaticus*, whose Cdt has a key role in carcinogenesis [147,148]. Some of these genotoxins can induce DBSs and G2/M cell cycle arrest [149,150,151]. The common structure comprises three subunits: a catalytic CdtB and two lectin-like subunits, which mediate host cell membrane adhesion and invasion [149].

CdtB exhibits PI-3,4,5-triphosphate phosphatase activity and DNase I-like structure and activity. These functions may explain its capacity to induce DSB and cell cycle arrest. Rapamycin alleviates CdtB genotoxicity, so its mechanism of action might be mediated by mTOR [148,149].

These toxins could also affect host gene expression and microbiota composition. Some studies have found an up-regulation of two carcinogenic pathways: peroxisome proliferator-activated receptors (PPAR) signaling pathway and calcium signaling pathway [148]. 

### 5.4. Bacteroides Fragilis Toxin (BFT)

BFT is a metalloprotease produced by Enterotoxigenic *B. fragilis*. The long-term presence of these bacteria, and therefore of BFT, may be related to the pathogenesis of familiar adenomatous polyposis (FAP) contributing to CRC development [152]. 

BFT is synthesized as a propeptide and processed into its active form before secretion. Once inside target cells, BFT promotes E-cadherins cleavage, stress response activation, cytokine secretion and increased proliferation [153,154].

E-cadherins degradation could be mediated by an unknown BFT surface protein receptor instead of BFT direct action as a metalloprotease [154]. Up-regulation of spermine oxidase (SMO) and the induction of cIAP2 (an antiapoptotic protein) may be some additional mechanisms. SMO is an inflammatory-inducible polyamine catabolic enzyme that promotes the generation of ROS and the induction of DNA damage [153].

### 5.5. Fusobacterium Nucleatum (Fn) Toxins

*Fusobacterium nucleatum* (*Fn*) is an anaerobic Gram-negative bacteria frequently found in CRC patients’ microbiota. There is a correlation between *Fn* infections with genetic and epigenetic changes related to poor CRC prognosis [155].

*Fn* adheres cell surface in order to invade and induce its oncogenic and inflammatory effects. FadA is a protein produced by *Fn* that binds to an 11-amino-acid region of the cell’s E-cadherin and promotes *Fn* attachment and invasion [156].

The mechanism underlying *Fn* oncogenic process may be mediated by ROS and pro-inflammatory factors. ROS could be responsible for the hypermethylation of CpG promoter islands and non-promoter hypomethylation of CpG regions leading to microsatellite instability and other epigenetic changes. Simultaneously, ROS and pro-inflammatory factors may induce DNA damage [155]. FadA could also contribute to inflammation via the β-catenin pathway [156].

Furthermore, *Fn* may disrupt NHEJ repair by downregulating KU70, a protein required to start the NHEJ process, while inducing DSB. Finally, the capacity to downregulate P27, a cyclin-dependent kinase inhibitor, increases cell proliferation and causes cell cycle arrest in S phase [157].

### 5.6. Klebsiella Oxytoca Enterotoxins

Antibiotic-associated hemorrhagic colitis (AAHC) is a disease caused by the expansion of colitogenic strains of *Klebsiella oxytoca* in some patients after antibiotics such as penicillin treatment [158]. Recently biofilms of *K. oxytoca* were identified in patients with CRC [159].

These bacteria present a gene cluster that encodes a non-ribosomal peptide assembly pathway that produces three secondary metabolites. Two of these metabolites, tilimycin (TM) and tilivalline (TV), present cytotoxic activity. The enzymes encoded by the gene cluster synthesize TV directly while TM requires the reaction between an imine intermediate metabolite of TV with indole [160].

TV is unable to bind to DNA or behave like a genotoxic agent by itself. TV induces microtubule-stabilization targeting tubulin and promoting tubulin-GTP state. This stabilization leads to a G2/M cell cycle arrest that is frequently resolved through multipolar anaphases. 

On the other hand, TM binds to and alkylates DNA directly. These lesions trigger a DDR in the host that could lead to the formation of adducts SSBs or DSB after the intervention of repair systems, arresting cells in G1 or S phase. Cells without Cockayne Syndrome group B protein (CSB), Cockayne Syndrome group A protein (CSA) and/or NER XPA resulted hypersensitive to TM, which could be explained by an essential role of TC-NER in the repair of TM induced-DNA lesions [160].

Both toxins may impair the intestinal barrier through two different mechanisms: increasing epithelial apoptosis and decreasing the expression of claudin-5 and claudin-8 proteins, contributing to tumour invasion and development [161].

## 6. Conclusions

The relationship between healthy habits, including diet and cancer, has been exhaustively researched. In this review, we envisioned this complex system emphasizing the direct or indirect roles of microbiota in DNA damage induction.

The diet and microbiota axis seems to be an indivisible factor. Microbiome’s metabolites may act as pro or anti-carcinogenic compounds depending on diet that in turn acts by remodeling microbiota composition itself. In this context, normal microbiota protects the epithelium barrier against harmful bacteria, inflammation, and DNA damage while diet-induced dysbiosis may lead to the opposite effects.

In conclusion, a greater understanding of DNA damage pathways induced by a diet-modified microbiota may lead to new approaches and treatments to decrease the risk of CRC development.
